# Effectiveness and Safety of S-1-Based Therapy Compared with 5-Fluorouracil-Based Therapy for Advanced Colorectal Cancer: A Meta-Analysis

**DOI:** 10.1155/2014/146530

**Published:** 2014-11-30

**Authors:** Jiaxiang Ye, Jiawei Chen, Lianying Ge, Aiqun Liu, Shaozhang Zhou

**Affiliations:** Department of Medical Oncology, The Cancer Institute, Affiliated Tumor Hospital of Guangxi Medical University, Nanning, Guangxi 530021, China

## Abstract

*Objectives.* The aim of our study was to compare the efficacy and safety of S-1-based therapy (SBT) versus 5-fluorouracil-based therapy (FBT) for advanced colorectal cancer (ACRC). *Methods.* A meta-analysis of all eligible randomized controlled trials (RCTs) was performed using RevMan 5.1.0 software. *Results.* A total of 1625 patients from twelve RCTs including 820 patients in the SBT group and 805 patients in the FBT group were available for analysis. The meta-analysis of overall survival (hazards ratio HR = 0.94, 95% CI = 0.80–1.10), progression-free survival (HR = 1.03, 95% CI = 0.91–1.18), and overall response rate (odds ratio OR = 1.23, 95% CI = 1.00–1.53) showed no statistical significance between SBT group and FBT group. The statistically significant differences in the meta-analysis indicated less incidence of graded 3-4 neutropenia (OR = 0.49, 95% CI = 0.35–0.68) and nausea/vomit (OR = 0.41, 95% CI = 0.23–0.72) in the SBT group, and there was no statistically significant difference in the incidence of grade 3-4 anemia, thrombocytopenia, leucopenia, diarrhea, and treatment-related deaths between two groups. *Conclusions.* SBT had similar efficacy and better safety than FBT and was an attractive alternative to FBT for patients of ACRC, but further investigations in different populations would be needed to confirm it.

## 1. Introduction

Despite advances in diagnosis and treatment, colorectal cancer remains the third leading cancer, with approximated 1,233,700 new cases and 608,700 deaths worldwide each year [1]. For the patients with advanced colorectal cancer (ACRC), acquiring curative therapy by surgery or radiotherapy is complex; therefore, systemic chemotherapy is the main effective treatment, which can prolong survival and enhance life quality of patients [2].

For many years, traditional continuous-infusion 5-fluorouracil (5-FU) in combination with leucovorin (LV) has been the backbone of palliative therapy for ACRC [2], and the combination of 5-FU and LV with either oxaliplatin (FOLFOX) or irinotecan (FOLFIRI) has been recognized as standard first-line therapies for ACRC [3]. However, administration of the traditional 5-FU regimens is time-consuming, uncomfortable, and inconvenient for the patients, because continuous infusion requires an indwelling central venous catheter with the associated increased risk of infection and thrombosis and regular hospital visits.

As substitute of 5-FU, S-1 (Taiho Pharmaceutical Company, Tokyo, Japan), an oral fluoropyrimidine, is a combined form of three pharmacological compounds (tegafur, gimeracil [CDHP], and oteracil potassium [Oxo]) at a molar ratio of 1 : 0.4 : 1. Tegafur is a prodrug that is mainly converted by liver enzyme cytochrome P450(CYP)2A6 to 5-FU, CDHP is an inhibitor of dihydropyrimidine dehydrogenase, which can prolong the half-life of 5-FU, and Oxo can reduce the toxic effects of 5-FU by inhibiting the phosphorylation of 5-FU to fluorouridine monophosphate in the gastrointestinal tract [4]. S-1-based therapy (SBT) has found to have similar efficacy and safety to 5-fluorouracil-based therapy (FBT) in the treatment of advanced gastric cancer (AGC) [5] and has been approved for the treatment of patients with AGC in japan.

The question that whether SBT has similar efficacy and safety to FBT in the treatment of ACRC is well worth exploring and studying. Recently, there have been a series of trials comparing S-1 with 5-FU in mono or combined therapy for ACRC [6–17]. However, single study may not be powered sufficiently to comprehensively assess the efficacy and safety of them, and so far there still has been not a meta-analysis of SBT versus FBT for ACRC. Consequently, we performed the present meta-analysis of all eligible studies to compare both treatment approaches and to evaluate their clinical efficacy and safety for patients of ACRC.

## 2. Methods

### 2.1. Literature Search

We conducted a comprehensive search by examining the PubMed, Embase, and the Cochrane Library Database for randomized controlled trials (RCTs) from inception to November 15, 2013, using various combinations of different terms “colorectal cancer,” “S-1,” “5-FU,” “randomized controlled trial,” and their synonyms or similar words (the “appendix” showed the search strategy of Embase, and the search strategy was also referred in other electronic databases). In addition, all abstracts from the American Society of Clinical Oncology (ASCO) conferences from inception to 2013 were also searched for relevant RCTs, and references cited in the identified articles were searched manually. The search was done without restriction on language.

### 2.2. Inclusion and Exclusion Criteria

Inclusion and exclusion criteria were delineated before the commencement of the literature search. Eligible studies were included in this meta-analysis if they met all the following criteria: (1) that participants were the patients of histologically confirmed, advanced, recurrent, or metastatic colorectal cancer and did not have severe basic diseases, (2) that only RCT was considered, (3) trials comparing SBT with FBT: mono or combined therapy of S-1 versus 5-FU and not confused by additional drugs or interventions (i.e., the experimental and control arms had difference only by S-1 and 5-FU components in the combination therapy). Accordingly, studies meeting the following criteria were excluded: (1) crossover studies and (2) the studies about loses visit rate >20%.

### 2.3. Data Extraction

Essential data was carefully extracted from all eligible studies independently by two investigators (Jia-Xiang Ye, Lian-Ying Ge), and discrepancies were finally resolved by consensus between the two authors (Jia-Xiang Ye, Lian-Ying Ge). From each study, we collected information on the following items: the first author's name, published year, country/region of origin, study design, characteristics of participants, interventions, and outcomes. When there were some updated results about the same study, we extracted the updated results. For the included studies with only abstract, we also acquired relevant clinical trial information by ClinicalTrials.gov Database (http://www.clinicaltrials.gov/).

### 2.4. Quality Assessment for Included Studies

Two authors (Jia-Xiang Ye, Lian-Ying Ge) assessed the quality of the eligible studies independently, with disagreements solved by a third author (Shao-Zhang Zhou) until agreement was obtained. With the guidance of the Cochrane Collaboration's tool for assessing risk of bias of RCTs (5.1.0) [20], we considered the following criteria to appraise the RCTs: random sequence generation, allocation concealment, binding of participants and personnel, binding of outcome assessment, incomplete outcome data, selecting reporting, and other bias. In all cases, high risk, low risk, or unclear risk was used to evaluate the risk of bias, and if insufficient detail was reported of what happened in the study, the judgment would usually be unclear risk of bias.

### 2.5. Statistical Analysis

Statistical analysis of the hazards ratio (HR) and 95% confidence interval (CI) for overall survival (OS) and progression-free survival (PFS), the odds ratio (OR) and 95% CI for overall response rate (ORR), disease control rate (DCR), one- or two-year survival rate (SR), and grade 3-4 adverse events (AEs) were calculated using RevMan 5.1.0 software. ORR was defined as the sum of partial and complete response rates according to the Response Evaluation Criteria in Solid Tumors, and the DCR was the sum of ORR and stable disease rate [21]. A fixed-effect model was used first, the *Q* test and *I* ^2^ statistic was performed to assess the heterogeneity, and *P* ≤ 0.1 or *I* ^2^ > 50% was considered as heterogeneity between studies. If the heterogeneity existed, sensitivity analysis or random-effect model was applied. Visual inspection of asymmetry in funnel plots was used to estimate the potential publication bias [22]. In order to supplement the funnel plot, Begg's test [23] and Egger's test [24] methods were performed using Stata version 12.0 software (Stata Corporation, College Station, TX).

## 3. Results

### 3.1. Study and Patient Characteristics

**Figure 1:**
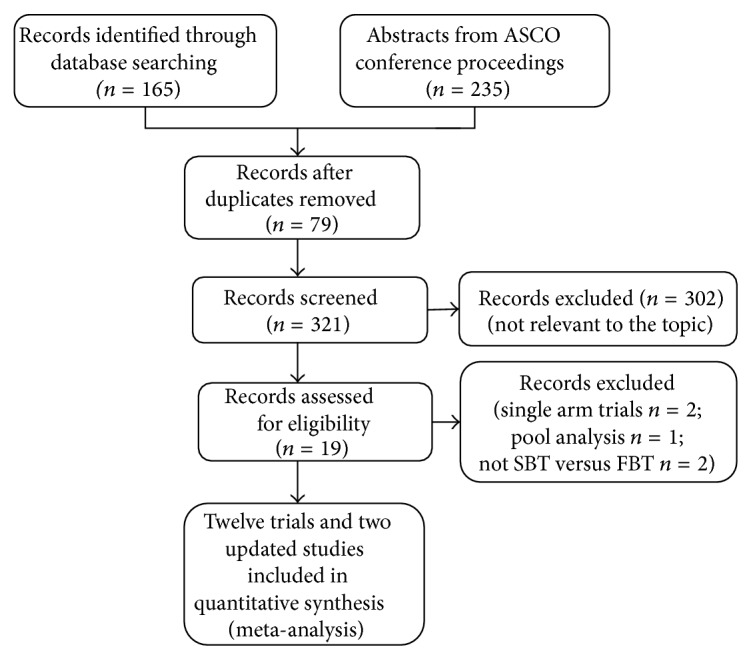
Flow chart displaying the process of study selection for the meta-analysis.

The search strategy yielded 400 records. Of these, 79 duplicates were eliminated and 302 articles were excluded due to irrelevancy by reviewing their titles and abstracts. The remaining 19 records were obtained to further determine eligibility. We ruled out another five articles: two articles due to single arm trials [25, 26], one article due to pooled analysis [27], and two trials not comparing SBT with FBT [28, 29]. So ten full texts [6, 7, 9, 11–17] and four abstracts [8, 10, 18, 19] were identified according to the inclusion criteria, of which the trial reported by Otsuji et al. [19] was the updated study of partial result of the trial reported by Ojima et al. [10], and the trial reported by Baba et al. [18] was the updated study of partial result of the trial reported by Muro et al. [9]. Thus, only twelve studies [6–17] assessing 1625 participants qualified to be included for this meta-analysis, as described in the flow chart (Figure 1).  Table 1 displayed the characteristics of these twelve individual trials with respect to author (year), country, demographic data, duration, intervention, outcome measure, and study design.

### 3.2. Quality of Eligible Studies

**Figure 2:**
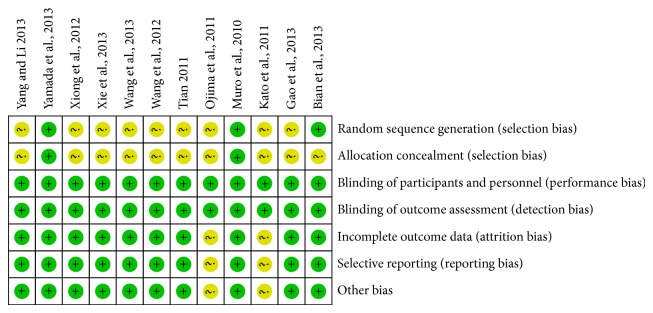
Risk of bias summary: review authors' judgements about each risk of bias item for each included study.

**Figure 3:**
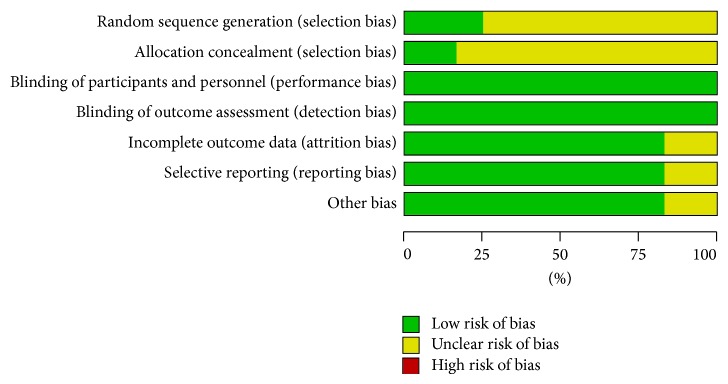
Risk of bias graph: review authors' judgements about each risk of bias item presented as percentages across all included studies.

All included studies undertook detailed assessments. All of the studies included the term “random,” but only three RCTs [6, 9, 16] reported the methods of random sequence generation, and only two RCTs [9, 16] reported the allocation concealment in detail. Moreover, although the three full texts [9, 12, 16] were open-label and other nine trials did not mention whether the blind method was adopted or not, these were unlikely to affect the quality assessment results. Two RCTs [9, 16] adequately described the missing data or missing reasons and took intention to treat analysis of all randomized patients. Eight RCTs [6, 7, 11–15, 17] reported complete outcome data. The ten RCTs had no other bias. Two trials [8, 10] were abstracts and included insufficient information regarding the outcome data, selective reporting and other bias (Figures 2 and 3).

### 3.3. Overall Survival

**Figure 4:**
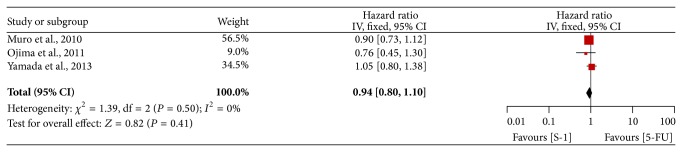
Forest plot of hazard ratio of overall survival.

Three of the four trials provided OS data [16, 18, 19]. The pooled HR of OS showed no significant difference between SBT and FBT yielding HR of 0.94 (95% CI 0.80–1.10) by using a fixed-effect model, and there was no significant heterogeneity across studies (*P* = 0.50, *I* ^2^ = 0%) (Figure 4).

### 3.4. One- or Two-Year Survival Rate

**Figure 5:**
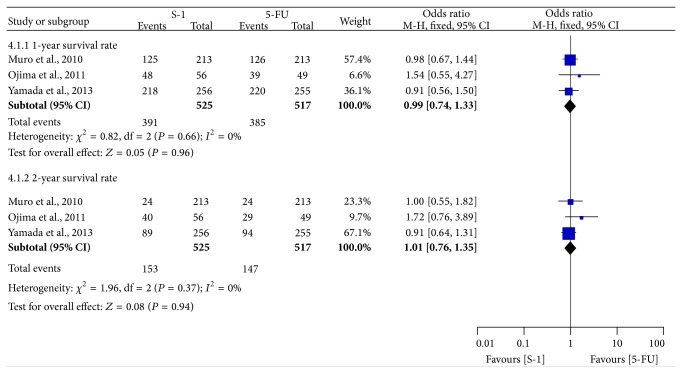
Forest plot of odds ratio of one- or two-year survival rate.

Three trials provided SR data [9, 10, 16, 19]. The pooled OR of one- or two-year SR showed there was no significant difference between SBT and FBT (1-year SR: OR = 0.99, 95% CI = 0.74–1.33; 2-year SR: OR = 1.01, 95% CI = 0.76–1.35) by using a fixed-effect model, with no heterogeneity across studies (1-year SR: *P* = 0.66, *I* ^2^ = 0%; 2-year SR: *P* = 0.37, *I* ^2^ = 0%) (Figure 5).

### 3.5. Progression-Free Survival

**Figure 6:**
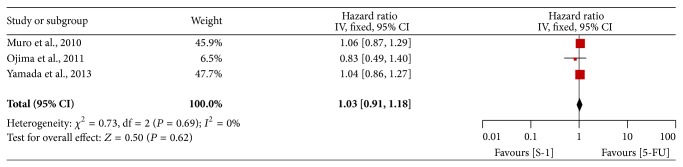
Forest plot of hazard ratio of progression-free survival.

Three trials provided PFS data [10, 16, 18]. The pooled HR of PFS showed there was no significant difference between SBT and FBT (HR = 1.03, 95% CI = 0.91–1.18), and the pooled HR of PFS was performed by using fixed-effects model, with no heterogeneity (*P* = 0.69, *I* ^2^ = 0%) (Figure 6).

### 3.6. Overall Response Rate or Disease Control Rate

**Figure 7:**
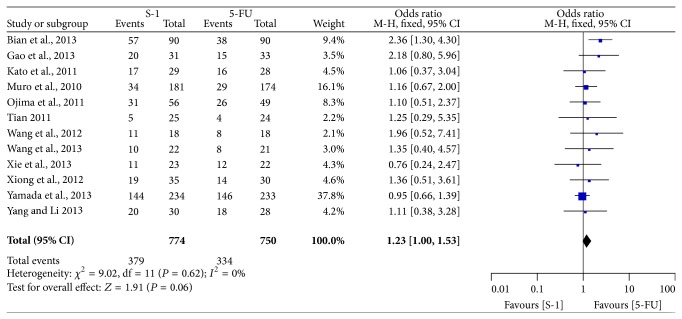
Forest plot of odds ratio of overall response rate.

**Figure 8:**
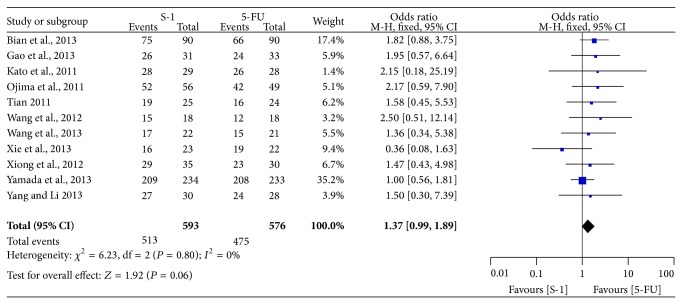
Forest plot of odds ratio of disease control rate.

All included studies provided the information on ORR [6–17]. As shown in Figure 7, the pooled OR of ORR in fixed-effect model was 1.23 (95% CI: 1.00–1.53) with little evidence of heterogeneity (*P* = 0.62, *I* ^2^ = 0%), which indicated there was no significant difference between SBT group and FBT group. Eleven trials reported DCR data [6–8, 10–17], the meta-analysis of the pooled data demonstrated that DCR was not different between the two groups (OR = 1.37, 95% CI = 0.99–1.89), and there was no heterogeneity across the trials (*P* = 0.80, *I* ^2^ = 0%) (Figure 8).

### 3.7. Safety

Results of graded 3 and 4 AEs analyses were shown in Table 2.

Neutropenia in hematologic toxicity: meta-analysis of four trials [9–11, 16] including 541 patients in the SBT group and 533 patients in the FBT group showed graded 3-4 neutropenia was less likely to happen in the SBT group (OR = 0.35, 95% CI = 0.27–0.47), and yet there was significant heterogeneity across these trials (*P* < 0.1, *I* ^2^ = 69%). Sensitivity analysis suggested that the trial reported by Yamada et al. [16] was the main source of heterogeneity. After removing this study, the heterogeneity was eliminated (*P* = 0.73, *I* ^2^ = 0.0%),  and the pooled result of the three trials applying fixed-effect model also showed that graded 3-4 neutropenia was significantly less likely to happen in patients of SBT than FBT (OR = 0.49, 95% CI = 0.35–0.68) (Figure 4(b)).

Leucopenia: meta-analysis of ten trials [6, 7, 9, 11–17] about graded 3-4 leucopenia, which included 734 patients in the SBT group and 726 patients in the FBT group, showed no significant difference between the two groups (OR = 0.75, 95% CI = 0.55–1.04), with no significant heterogeneity across studies (*P* = 0.28, *I* ^2^ = 18%).

Anemia: nine trials [6, 7, 9, 11, 12, 14–17] reported graded 3-4 anemia assessing 1417 participants (SBT, *n* = 712; FBT, *n* = 705), meta-analysis of the pooled data showed no significant difference between the two groups (OR = 1.33, 95% CI = 0.83–2.15), and there was no significant heterogeneity across studies (*P* = 0.40, *I* ^2^ = 3%).

Thrombocytopenia: the pooled OR of graded 3-4 thrombocytopenia of nine trials [6, 7, 9, 12–17] assessing 1411 participants (SBT, *n* = 709; FBT, *n* = 702) showed no significant difference between the two groups (OR = 1.05, 95% CI = 0.51–2.15), and there was no significant heterogeneity across studies (*P* = 0.28, *I* ^2^ = 21%).

Diarrhea in nonhematologic toxicity: all included studies provided the information on grade 3-4 diarrheas. Meta-analysis of twelve trials showed a significant heterogeneity across the trials (*P* < 0.10, *I* ^2^ = 64%). Sensitivity analysis did not find the main source of heterogeneity. So meta-analysis of twelve trials assessing 1625 participants (SBT, *n* = 820; FBT, *n* = 805) in random-effect model showed no significant difference between the two groups (OR = 1.25, 95% CI = 0.58–2.69).

Nausea/vomit: meta-analysis of ten trials [6–9, 12–17] assessing 1471 participants (SBT, *n* = 739; FBT, *n* = 732) in fixed-effect model showed no significant difference between the two groups (OR = 0.41, 95% CI = 0.23–0.72), with no heterogeneity (*P* = 0.56, *I* ^2^ = 0%).

Stomatitis: nine trials [6–9, 12, 13, 15–17] reported graded 3-4 stomatitis assessing 1426 participants (SBT, *n* = 716; FBT, *n* = 710), meta-analysis of the pooled data showed no significant difference between the two groups (OR = 2.21, 95% CI = 0.83–5.88), and there was no significant heterogeneity across studies (*P* = 0.24, *I* ^2^ = 28%).

Treatment-related death (TRD): data on the TRD were available for five trials [8, 9, 11, 16, 17] including 1087 participants (SBT, *n* = 545; FBT, *n* = 542) in the meta-analysis. The pooled OR of five trials showed TRD was not significantly different between the two groups (OR = 0.72, 95% CI = 0.24–2.19), and there was no heterogeneity among the studies (*P* = 0.71, *I* ^2^ = 0%).

### 3.8. Publication Bias

**Figure 9:**
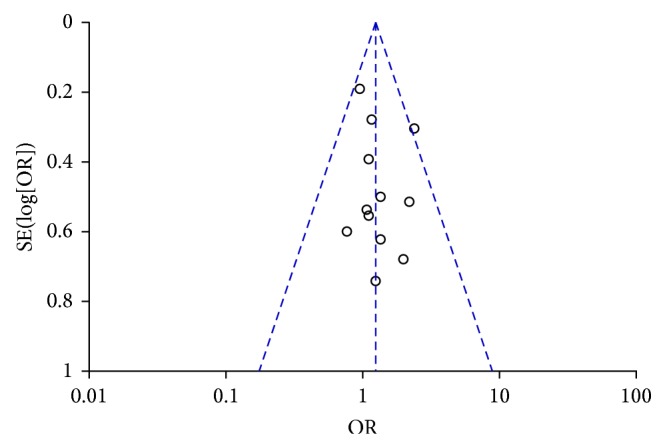
Funnel plot.

The visual inspection of the funnel plots suggested a roughly symmetrical distribution for the study (Figure 9), which showed no evidence of publication bias. Moreover, according to Begg's test (*P* = 0.49) and Egger's test (*P* = 0.40), publication bias was also not found.

## 4. Discussion

To our knowledge, this was the first meta-analysis to evaluate the efficacy and safety of SBT versus FBT for ACRC. A total of 1625 patients from twelve RCTs including 820 patients in the SBT group and 805 patients in the FBT group were analyzed. With respect to ORR, DCR, and one- or two-year SR, our meta-analysis showed no significant difference between SBT and FBT group, which suggested that SBT was noninferior to FBT for the patients of ACRC. And the pooled analysis showed that SBT had similar PFS and OS to FBT as well. All these results indicated SBT had similar efficacy to FBT for patients of ACRC.

For safety profile, Our meta-analysis showed grade 3-4 toxicity such as anemia, leucopenia, thrombocytopenia, diarrhea, and TRD was similar between two groups, but the graded 3-4 neutropenia (OR = 0.49, 95% CI = 0.35–0.68) and nausea/vomit (OR = 0.41, 95% CI = 0.23–0.72) were less likely to happen in the SBT group than in the FBT group. Thus, compared with FBT, SBT could reduce some toxicity for patients of ACRC. Owing to significant heterogeneity in grade 3-4 neutropenia, sensitivity analysis was performed to find the contributors of heterogeneity, the trial reported by Yamada et al. [16], and the main source of heterogeneity might be attributable to the different therapy regimens; for instance, the therapy regimen of the trial reported by Yamada et al. had a biological targeted drug (bevacizumab), but the therapy regimens of the other studies of meta-analysis did not include bevacizumab. Regarding grade 3-4 diarrhea, a sensitivity analysis was also performed, but the factors contributing to the heterogeneity could not be identified; these might be associated with variations in age, performance status of patients, dose, and the regimen of therapy between the trials. Thus, a random-effect model was applied to compensate for this.

Capecitabine is another oral fluorouracil-derivative drug designed to simulate a continuous intravenous infusion of 5-FU [30], whose efficacy and safety had been found to be at least equivalent to that of 5-FU for ACRC by many studies [31]. And yet, in 2012 the phase 3 trial reported by Hong et al. showed the noninferiority of oxaliplatin and S-1(SOX) versus oxaliplatin and capecitabine (XELOX) for ACRC, with median PFS of 8.5 months in the SOX group and 6.7 months in the XELOX group (HR = 0.79, 95% CI 0.60–1.04), and manageable toxicities in both groups [28]. Later the results of a randomized phase II study also suggested that both SOX and XELOX regimens were active and were well tolerated regimens in patients with ACRC [29]. These studies suggested indirectly that S-1-based chemotherapy (SBCT) could be regarded as an alternate chemotherapy strategy to 5-FU-based chemotherapy (FBCT) for the patients of ACRC.

Recently 5-FU-based chemotherapy plus bevacizumab (FBCT + Bev) has been widely used for first-line treatment of ACRC [32, 33], but S-1-based chemotherapy plus bevacizumab (SBCT + Bev) is still at the research stage. In 2013, Yamada et al. performed a randomised phase 3 trial, showing that SBCT + Bev was noninferior to FBCT + Bev in the first-line treatment for ACRC, with median PFS of 11.7 months in SBT group and 11.5 months in FBT group, and AEs in both groups were tolerable [16]. Moreover, the median PFS of SBCT + Bev was about 2 months longer than that of similar SBCT without bevacizumab in the treatment of patients with similar characteristics in the phase III trial reported by Hong et al. [28]. What is more, SBCT + Bev did not require a long infusion process and reduced the inconvenience for patients. Therefore, compared with FBCT + Bev, SBCT + Bev provided a great advantage and was a promising regimen for patients of ACRC.

Of note, S-1 had different optimal doses and safety among patients in different regions and populations. Since the gene polymorphic variants of liver enzyme CYP2A6 converting tegafur to 5-FU were less frequent in the Caucasians than in East Asians, the tolerable dose of S-1 might be lower for Caucasian patients than for patients in East Asia; in other words, toxic effects were more common in Caucasian patients exposed to the same dose of S-1 [34, 35]. Besides, variation in creatinine clearance was associated with S-1 toxicity as well, which meant dose adjustment should be considered when S-1 was used for patients with compromised renal function [16]. Thus, the use of different dose of S-1 should be clarified for suitable population.

There were several limitations in this meta-analysis. First, since all the studies included in this analysis were from East Asia, the results needed confirmation in other countries. Second, the quality of the studies was not considered to be high, only three RCTs [6, 9, 16] reported the methods of random sequence generation, and two RCTs [9, 16] reported the allocation concealment in detail. More RCTs with improved methodological quality should be provided to update this study. Third, two studies in the meta-analysis were abstracts [8, 10], whose incomplete information might potentially limit estimate of SBT effects. Moreover, information of each individual patient for each trial was not obtained to make comprehensive analysis. Last, there was one heterogeneous result about AEs.

In summary, our meta-analysis indicated that SBT had similar efficacy and better safety than FBT for patients of ACRC. Given its advantages of simplicity and convenience to administer, SBT would be an attractive alternative to FBT for patients of ACRC, especially for outpatients. Owing to the variation of S-1 tolerance in different regions and populations, further high-quality RCTs and different population studies in future would be needed to confirm it.

## Figures and Tables

**Table 1:** Main characteristics of the studies included in the meta-analysis.

Study	Country	Duration	Number of Patients (A/B)	Regimen (group A and group B)	Outcome measures	Study design
			PS (score)			
Muro et al. 2010 [9], Baba et al. 2011 [18]	Japan	Jan. 2006–Jan. 2008	213/213 0-1	A: irinotecan 125 mg/m^2^ d1d15, S-1 40–60 mg (according to body surface area) bid d1–14, q4 w B: LV 200 mg/m^2^ d1, irinotecan 150 mg/m^2^ d1, 5-FU 400 mg/m^2^ d1 and 2400 mg/m^2^ civ 46 h, q2 w	ORR, PFS, OS, toxicities	Randomized phase II/III study

Kato et al. 2011 [8]	Japan	July 2007–Mar. 2010	30/30 0-1	A: irinotecan 150 mg/m^2^ d1, S-1 80 mg/m^2^ d3–16, bevacizumab 7.5 mg/kg, q3 w B: irinotecan 150 mg/m^2^ d1, LV 200 mg/m^2^ d1, 5-FU 400 mg/m^2^ d1 and 2400 mg/m^2^ civ 46 h, bevacizumab 5 mg/kg, q2 w	ORR, toxicities	Randomized pilot study

Ojima et al. 2011 [10], Otsuji et al. 2012 [19]	Japan	July 2008–July 2009	56/49 0-1	A: S-1 40–60 mg bid d1–7, oral LV 25 mg bid d1–7, oxaliplatin 85 mg/m^2^ d1, q2 w B: oxaliplatin 85 mg/m^2^ d1, LV 200 mg/m^2^ d1, 5-FU 400 mg/m^2^ d1 and 2400 mg/m^2^ civ 46 h, q2 w	ORR, PFS, SR, OS, toxicities	Randomized phase II trial

Yamada et al. 2013 [16]	Japan	Feb. 2009–Mar. 2011	256/255 0-1	A: oxaliplatin 130 mg/m^2^ d1, S-1 40–60 mg (according to body surface area) bid d1–14, bevacizumab 7.5 mg/kg, q3 w B: bevacizumab 5 mg/kg, LV 200 mg/m^2^ d1, oxaliplatin 85 mg/m^2^ d1, 5-FU 400 mg/m^2^ d1 and 2400 mg/m^2^ civ 46 h, q2 w	ORR, PFS, OS, toxicities	Randomized phase III trial

Yang and Li 2013 [17]	China	Jan. 2010–Jun. 2012	30/28 0–2	A: irinotecan 180 mg/m^2^ d1, S-1 80 mg/m^2^ d1–14, q3 w B: irinotecan 180 mg/m^2^ d1, LV 200 mg/m^2^ d1, 5-FU 400 mg/m^2^ d1 and 2400 mg/m^2^ civ 46 h, q2 w	ORR, toxicities	Randomized controlled trial

Xie et al. 2013 [14]	China	Mar. 2009–Sep. 2012	23/22 0–2	A: S-1 80 mg/m^2^ d1–14, oxaliplatin 100 mg/m^2^ d1, q3 w B: oxaliplatin 100 mg/m^2^ d1, LV 400 mg/m^2^ d1, 5-FU 400 mg/m^2^ d1 and 2400 mg/m^2^ civ 46 h, q2 w	ORR, TTP, toxicities	Randomized controlled trial

Wang et al. 2013 [13]	China	Mar. 2008–Dec. 2012	22/21 0–2	A: S-1 80 mg/m^2^ d1–14, oxaliplatin 130 mg/m^2^ d1, q3 w B: oxaliplatin 130 mg/m^2^ d1, LV 200 mg/m^2^ d1, 5-FU 400 mg/m^2^ d1 and 2400 mg/m^2^ civ 46 h, q3 w	ORR, toxicities	Randomized controlled trial

Wang et al. 2012 [12]	China	NA	18/18 ≥70^a^	A: S-1 80 mg/m^2^ d1–14, oxaliplatin 85 mg/m^2^ d1, q4 w B: oxaliplatin 85 mg/m^2^ d1, LV 200 mg/m^2^ d1, 5-FU 300 mg/m^2^ d1–5, q4 w	ORR, TTP, MST, toxicities	Randomized controlled trial

Tian 2011 [11]	China	Jun 2009–May 2011	25/24 0-1	A: irinotecan 125 mg/m^2^ d1d15, S-1 40–60 mg (according to body surface area) bid d1–14, q4 w B: irinotecan 150 mg/m^2^ d1, LV 200 mg/m^2^ d1, 5-FU 400 mg/m^2^ d1 and 2400 mg/m^2^ civ 46 h, q2 w	ORR, TTP, toxicities	Randomized controlled trial

Bian et al. 2013 [6]	China	Jan. 2011–Dec. 2012	90/90 0-1	A: S-1 80 mg/m^2^ d1–14, oxaliplatin 85 mg/m^2^ d1, q4 w B: oxaliplatin 85 mg/m^2^ d1, LV 200 mg/m^2^ d1, 5-FU 300 mg/m2 d1–5, q4 w	ORR, toxicities	Randomized controlled trial

Xiong et al. 2012 [15]	China	Mar. 2010–Jun 2011	35/30 ≥70^a^	A: S-1 80 mg/m^2^ d1–14, oxaliplatin 100 mg/m^2^ d1, q3 w B: oxaliplatin 100 mg/m^2^ d1, LV 400 mg/m^2^ d1, 5-FU 400 mg/m^2^ d1 and 2400 mg/m^2^ civ 46 h, q2 w	ORR, toxicities	Randomized controlled trial

Gao et al. 2013 [7]	China	Jan. 2010–Jun 2012	31/33 ≥70^a^	A: irinotecan 100 mg/m^2^ d1d8, S-1 40 mg/m^2^ d1–14, q3 w B: irinotecan 180 mg/m^2^ d1, LV 200 mg/m^2^ d1, 5-FU 400 mg/m^2^ iv d1d2 and 600 mg/m^2^ civ 22 h, d1d2, q2 w	ORR, toxicities	Randomized controlled trial

Note: LV: leucovorin; 5-FU: 5-fluorouracil; PS: performance status; ORR: overall response rate; OS: overall survival; PFS: progression-free survival; MST: median survival time; TTP: time to progression; SR: survival rate; d1–14: days 1–14; q3 w: every 3 weeks; q4 w: every 4 weeks; q6 w: every 6 weeks; bid: twice a day; NA: not available; civ: continuous intravenous infusion; ^a^Karnofsky method.

**Table 2:** Outcome of toxicity meta-analysis comparing S-1 versus 5-FU in advanced colorectal cancer.

Toxicity	Trials	SBT	FBT	Heterogeneity		OR (95% CI)	Model	*P* value
				*P* value	*I* ^2^ (%)			
Grade 3-4 neutropenia	3	96/291	143/284	0.73	0	0.49 [0.35, 0.68]	Fixed	<0.01
Grade 3-4 leucopenia	10	79/734	100/726	0.28	18	0.75 [0.55, 1.04]	Fixed	0.08
Grade 3-4 anemia	9	41/712	31/705	0.40	3	1.33 [0.83, 2.15]	Fixed	0.24
Grade 3-4 thrombocytopenia	9	15/709	14/702	0.28	21	1.05 [0.51, 2.15]	Fixed	0.89
Grade 3-4 diarrhea	12	92/820	49/805	<0.1	64	1.25 [0.58, 2.69]	Random	0.57
Grade 3-4 nausea/vomit	10	15/739	38/732	0.56	0	0.41 [0.23, 0.72]	Fixed	<0.01
Grade 3-4 stomatitis	9	11/716	4/710	0.24	28	2.21 [0.83, 5.88]	Fixed	0.11
Treatment-related death	5	4/545	6/542	0.71	0	0.72 [0.24, 2.19]	Fixed	0.57

Notes: NA: not available; OR: odds ratio; CI: confidence interval; FBT: 5-fluorouracil-based therapy; SBT: S-1-based therapy.

## Appendix

EMBASE search terms:

(1) “colorectal”: ab, ti OR “rectal”: ab, ti OR “colon”: ab, ti OR “intestinal”: ab, ti OR “colorectal carcinoma”/exp OR “colorectal tumor”/exp;
(2) “tegafur gimeracil oteracil potassium”: ab, ti OR “S-1”: ab, ti OR “TS-1”: ab, ti OR “gimeracil plus oteracil potassium plus tegafur”/exp;
(3) “fluorouracil”: ab, ti OR “5-FU”: ab, ti OR “fluorouracil”/exp;
(4) “trial”: ab, ti OR “randomized”: ab, ti OR “randomly”: ab, ti OR “random”: ab, ti OR “groups”: ab, ti OR “placebo”: ab, ti OR “randomized controlled trial”/exp OR “randomized controlled trial (topic)”/exp;
(5) #1 AND #2 AND #3 AND #4.
